# Supplementary material for: Explainable artificial intelligence to identify follicles that optimize clinical outcomes during assisted conception
Source: Nat Commun. 2025 Jan 8;16:296. doi: 10.1038/s41467-024-55301-y (PMC11711444; doi:10.1038/s41467-024-55301-y)
Supplement: Supplementary file 1 — Supplementary information [file 41467_2024_55301_MOESM1_ESM.pdf]

Supplementary Table 1: Model performance metrics per clinic for all oocytes (n=19,082) and metaphase-II (MII) oocytes (n=14,140) as outcome variables. For each model, the mean absolute error (MAE), coefficient of determination (R^2), and median absolute error (MedAE) are reported per clinic when serving as the test set. Similarly, for the outcome variables all oocytes and MII oocytes, the minimum, lower quartile (LQ), median, mean, upper quartile (UQ), maximum, interquartile range (IQR), and standard deviation (SD) measures are reported. The final row represents data summaries for the whole dataset.

Clinic	Outcome	MAE	R^2	MedAE	Min	LQ	Median	Mean	UQ	Max	IQR	SD
A	All oocytes	3.84	0.60	2.61	1.00	8.00	13.00	14.32	19.00	76.00	11.00	8.93
	MII oocytes	3.47	0.50	2.26	0.00	6.00	9.00	10.87	15.00	54.00	9.00	7.26
B	All oocytes	3.64	0.23	2.82	1.00	7.00	11.00	11.14	15.00	36.00	8.00	5.56
	MII oocytes	3.16	0.12	2.61	0.00	4.00	7.00	7.35	10.00	28.00	6.00	4.30
C	All oocytes	4.08	0.54	2.70	1.00	6.00	10.00	12.78	17.25	71.00	11.25	9.37
	MII oocytes	3.76	0.45	2.50	0.00	4.00	8.00	9.87	13.00	58.00	9.00	7.80
D	All oocytes	4.37	0.18	3.02	1.00	7.00	11.00	11.80	16.00	45.00	9.00	6.79
	MII oocytes	4.53	0.15	3.39	0.00	4.00	8.00	8.50	12.00	41.00	8.00	6.64
E	All oocytes	4.10	0.41	2.56	1.00	7.00	11.00	12.62	16.00	70.00	9.00	8.45
	MII oocytes	3.58	0.34	2.27	0.00	5.00	8.00	10.11	14.00	68.00	9.00	6.94
F	All oocytes	3.88	0.49	2.73	1.00	7.00	11.00	12.47	16.00	74.00	9.00	8.11
	MII oocytes	3.31	0.38	2.28	0.00	5.00	8.00	8.74	11.00	33.00	6.00	5.78
G	All oocytes	3.67	0.59	2.58	0.00	7.00	11.00	12.81	17.00	70.00	10.00	8.04
	MII oocytes	3.33	0.53	2.38	0.00	5.00	9.00	10.10	13.00	54.00	8.00	6.68
H	All oocytes	3.94	0.51	2.70	1.00	8.00	12.00	13.92	18.00	86.00	10.00	8.23
	MII oocytes	3.66	0.42	2.44	0.00	6.00	10.00	11.38	15.00	65.00	9.00	6.95
I	All oocytes	3.28	0.48	2.41	0.00	6.00	9.00	10.12	13.00	42.00	7.00	6.19
	MII oocytes	3.59	0.29	2.62	0.00	3.00	6.00	7.40	10.00	37.00	7.00	5.77
J	All oocytes	3.72	0.36	2.68	1.00	7.00	11.00	12.36	16.00	35.00	9.00	6.31
	MII oocytes	3.32	0.26	2.57	0.00	5.00	8.00	8.86	12.00	30.00	7.00	5.17
K	All oocytes	3.83	0.53	2.72	1.00	9.00	13.00	14.27	19.00	62.00	10.00	7.86
	MII oocytes	3.52	0.44	2.61	0.00	6.00	10.00	10.91	14.50	48.00	8.50	6.57
All	All oocytes	3.85	0.45	2.68	0.00	7.00	11.00	12.54	16.00	86.00	9.00	7.78
	MII oocytes	3.60	0.35	2.59	0.00	5.00	8.00	9.62	13.00	68.00	8.00	6.59

Supplementary Table 2: Hyperparameters that were chosen to tune relevant to the HistGradientBoostingRegressor model using Bayesian optimization.

Hyperparameter	Search space
max_iter	(500, 5000)
learning_rate	(0.0001, 0.1)
l2_regularization	(0.0, 1.0)
min_samples_leaf	(5, 20)
loss	(squared error, absolute error, Poisson)

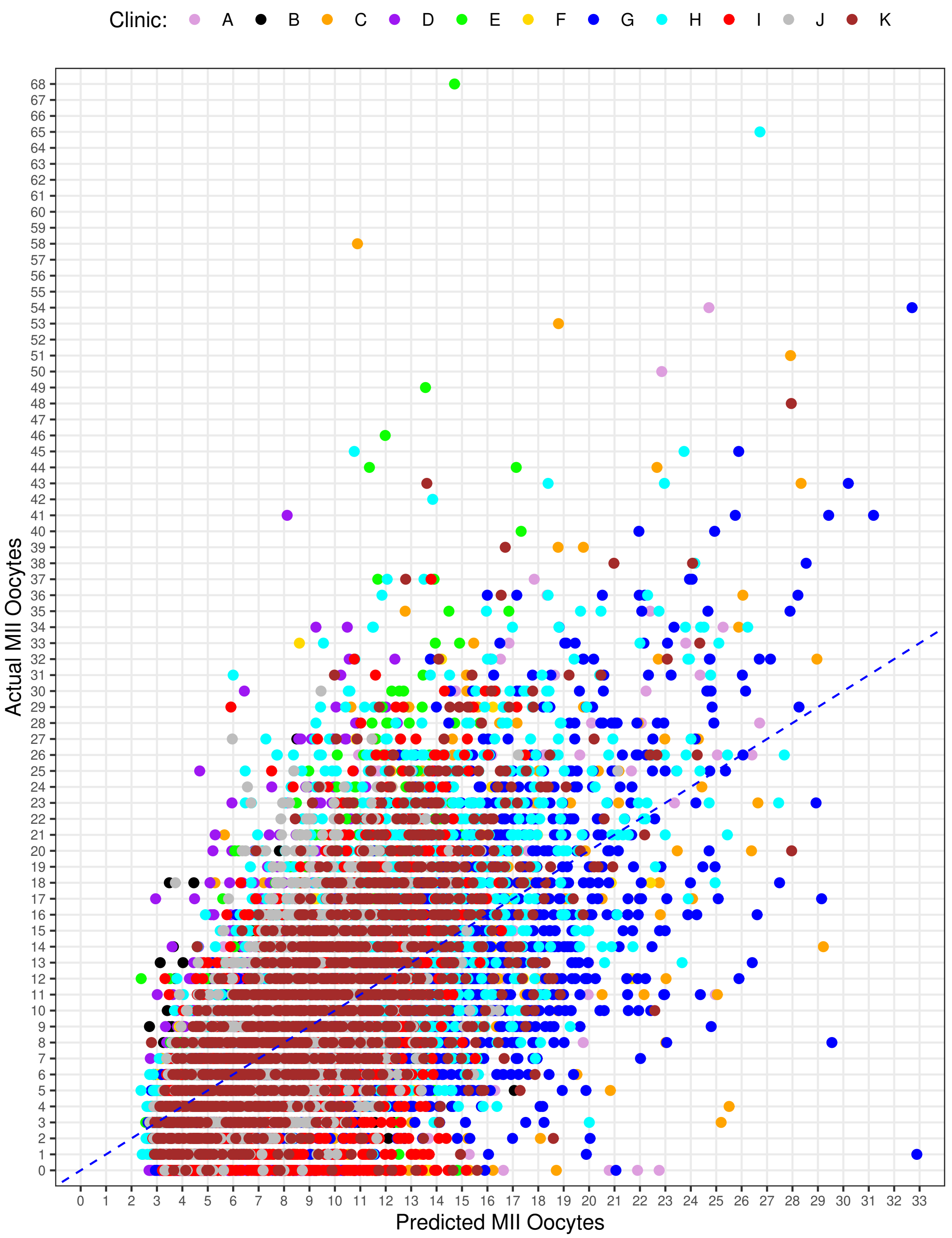

Supplementary Figure 1: Predicted versus actual values for the metaphase-II (MII) oocyte model (n=14,140 patients) colored by clinic label, A-K.
