# Supplementary material for: Explainable artificial intelligence to identify follicles that optimize clinical outcomes during assisted conception
Source: Nat Commun. 2025 Jan 8;16:296. doi: 10.1038/s41467-024-55301-y (PMC11711444; doi:10.1038/s41467-024-55301-y)
Supplement: Supplementary file 2 — Reporting Summary [file 41467_2024_55301_MOESM2_ESM.pdf]

nature portfolio

Corresponding author(s): Ali Abbara
Last updated by author(s): Dec 4, 2024

Reporting Summary

Nature Portfolio wishes to improve the reproducibility of the work that we publish. This form provides structure for consistency and transparency in reporting. For further information on Nature Portfolio policies, see our Editorial Policies and the Editorial Policy Checklist.

Statistics

For all statistical analyses, confirm that the following items are present in the figure legend, table legend, main text, or Methods section.

n/a	Confirmed	
☐	☒	The exact sample size (*n*) for each experimental group/condition, given as a discrete number and unit of measurement
☐	☒	A statement on whether measurements were taken from distinct samples or whether the same sample was measured repeatedly
☐	☒	The statistical test(s) used AND whether they are one- or two-sided<br>*Only common tests should be described solely by name; describe more complex techniques in the Methods section.*
☐	☒	A description of all covariates tested
☐	☒	A description of any assumptions or corrections, such as tests of normality and adjustment for multiple comparisons
☐	☒	A full description of the statistical parameters including central tendency (e.g. means) or other basic estimates (e.g. regression coefficient) AND variation (e.g. standard deviation) or associated estimates of uncertainty (e.g. confidence intervals)
☐	☒	For null hypothesis testing, the test statistic (e.g. *F*, *t*, *r*) with confidence intervals, effect sizes, degrees of freedom and *P* value noted<br>*Give P values as exact values whenever suitable.*
☒	☐	For Bayesian analysis, information on the choice of priors and Markov chain Monte Carlo settings
☒	☐	For hierarchical and complex designs, identification of the appropriate level for tests and full reporting of outcomes
☐	☒	Estimates of effect sizes (e.g. Cohen's *d*, Pearson's *r*), indicating how they were calculated

Our web collection on statistics for biologists contains articles on many of the points above.

Software and code

Policy information about availability of computer code

Data collection	Data was collated and extracted from IDEAS (Mellowood Medical Inc), which is a specialized commercial electronic medical record database system for fertility practices. Collected data are private (see data availability statement).
Data analysis	Machine learning analyses were conducted using standard open source packages on Python 3.6 (e.g., including 'scikit-learn' and 'shap'). Further statistical analysis were performed on R version 4.3.3. Graphing and common statistical analyses were carried out using GraphPad Prism 10.3. More detail regarding the code can be explored further from the documentation of the relevant underlying packages described in the manuscript.

For manuscripts utilizing custom algorithms or software that are central to the research but not yet described in published literature, software must be made available to editors and reviewers. We strongly encourage code deposition in a community repository (e.g. GitHub). See the Nature Portfolio guidelines for submitting code & software for further information.

Data

Policy information about availability of data

All manuscripts must include a data availability statement. This statement should provide the following information, where applicable:

- Accession codes, unique identifiers, or web links for publicly available datasets
- A description of any restrictions on data availability
- For clinical datasets or third party data, please ensure that the statement adheres to our policy

The data that support the findings of this study were obtained from TFP Fertility under a legal non-disclosure agreement and non-commercial terms. Due to the

nature of these agreements, the data are not publicly available and cannot be shared by the researchers with third parties. Any requests for access to the raw data should be directed to the Business Intelligence team at TFP Fertility (bi-support@tfp-fertility.com). Requests should specify the principal investigator, a description of the proposed study, and the reason for data access. The proposed ML models and statistical pipelines openly available in the code are not specific to the datasets used in this study. A source data file is provided with this paper.

Research involving human participants, their data, or biological material

Policy information about studies with human participants or human data. See also policy information about sex, gender (identity/presentation), and sexual orientation and race, ethnicity and racism.

Reporting on sex and gender	Findings apply only to patients with female sex at birth (self-reported) who have undergone in vitro fertilization (IVF) treatment.
Reporting on race, ethnicity, or other socially relevant groupings	Race, ethnicity, or other socially relevant groupings were not analysed in the present study.
Population characteristics	Treatment-naive patients who had a transvaginal ultrasound scan presenting at least three follicles >10mm on the same day as the day of trigger administration when undergoing IVF treatment, and had the number of oocytes collected recorded. This cohort was 18-49 years of age at the time of treatment with an average body mass index (BMI) of 25.0 and an antral follicle count of 17.4.
Recruitment	Retrospective database analysis which included only treatment-naive patients who had a transvaginal ultrasound scan presenting at least three follicles >10mm on the same day as the day of trigger administration.
Ethics oversight	Data included in this manuscript were obtained from retrospective data carried out following the sponsorship of the institutional review board and approval by the Health Research Authority (23/HRA/2849). All ART clinics were under a license from the Human Fertilisation and Embryology Authority (UK) or the Ministry of Health (Poland).

Note that full information on the approval of the study protocol must also be provided in the manuscript.

Field-specific reporting

Please select the one below that is the best fit for your research. If you are not sure, read the appropriate sections before making your selection.

☒ Life sciences ☐ Behavioural & social sciences ☐ Ecological, evolutionary & environmental sciences

For a reference copy of the document with all sections, see nature.com/documents/nr-reporting-summary-flat.pdf

Life sciences study design

All studies must disclose on these points even when the disclosure is negative.

Sample size	Retrospectrive treatment data extracted from 19,082 treatment-naive patients.
Data exclusions	Patients were included if they were treatment-naive and had a transvaginal ultrasound scan presenting at least three follicles >10mm on the same day as the day of trigger administration when undergoing IVF treatment, with the number of oocytes collected recorded.
Replication	Methods needed to replicate the study and data analysis are described in the methods.
Randomization	Data was retrieved across 11 separate clinics. Modelling was achieved using a leave-one-clinic-out cross-validation method (noted as 'internal-external validation') and described in detail in the manuscript.
Blinding	Retrospective analysis and therefore not applicable.

Reporting for specific materials, systems and methods

We require information from authors about some types of materials, experimental systems and methods used in many studies. Here, indicate whether each material, system or method listed is relevant to your study. If you are not sure if a list item applies to your research, read the appropriate section before selecting a response.

Materials & experimental systems

n/a	Involved in the study
☒	☐ Antibodies
☒	☐ Eukaryotic cell lines
☒	☐ Palaeontology and archaeology
☒	☐ Animals and other organisms
☒	☐ Clinical data
☒	☐ Dual use research of concern
☒	☐ Plants

Methods

n/a	Involved in the study
☒	☐ ChIP-seq
☒	☐ Flow cytometry
☒	☐ MRI-based neuroimaging

Plants

Seed stocks	*Report on the source of all seed stocks or other plant material used. If applicable, state the seed stock centre and catalogue number. If plant specimens were collected from the field, describe the collection location, date and sampling procedures.*
Novel plant genotypes	*Describe the methods by which all novel plant genotypes were produced. This includes those generated by transgenic approaches, gene editing, chemical/radiation-based mutagenesis and hybridization. For transgenic lines, describe the transformation method, the number of independent lines analyzed and the generation upon which experiments were performed. For gene-edited lines, describe the editor used, the endogenous sequence targeted for editing, the targeting guide RNA sequence (if applicable) and how the editor was applied.*
Authentication	*Describe any authentication procedures for each seed stock used or novel genotype generated. Describe any experiments used to assess the effect of a mutation and, where applicable, how potential secondary effects (e.g. second site T-DNA insertions, mosiacism, off-target gene editing) were examined.*
